# Access to formal education for the San community in Tsholotsho, Zimbabwe: challenges and prospects

**DOI:** 10.1016/j.heliyon.2020.e04470

**Published:** 2020-07-17

**Authors:** Keith Phiri, Sibonokuhle Ndlovu, Thulani Dube, Douglas Nyathi, Cornelias Ncube, Nhlalo Tshuma

**Affiliations:** a Department of Development Studies, Faculty of Humanities and Social Sciences, Lupane State University, Zimbabwe; b Faculty of Humanities and Social Sciences, Lupane State University, Zimbabwe

**Keywords:** Education, Ethnography, Marginalization, Poverty, San community

## Abstract

This article examines the myriad of challenges faced by primary and secondary school going children amongst the San community in their quest to attain formal education in rural Tsholotsho, Zimbabwe. Using a mixed method approach, the study utilised focus group discussions from selected primary and secondary schools, key informant interviews with headmasters and teachers and also survey questionnaires supported by an ethnographic research design. It emerged from the study that long distance travelled to school, abject poverty, dilapidated infrastructure and the perceived negative attitude towards education has had telling effects on the marginalised San community. The hindrances to attaining formal education has tended to solidify the existing stereotypes, prejudices and social labels against the San community by other ethnic groups as “separatists”, “non-conformist” and at worst “primitive” in as far as participating in modern and mainstream development is concerned. The article suggests that there is a need for a paradigm shift in attitude and behaviour of development agents and actors in their policy positions and their interventions to the San community. Formal education for the San community will be hard to achieve as long as institutions perceive them as subaltern citizens, who are a nuisance.

## 1 Introduction: who are the San people?

The San, also known as the Bushmen, are thought to be the oldest residents of Africa who have resided in Southern Africa for more than 20 000 years (Mitchell, 2013, cited in Hitchcock et al., 2016). In Zimbabwe, for instance, the San self-identify as the true indigenous people of the country ahead of other groups (IWGIA, 2014). In Southern Africa, the San people are found in seven countries; namely, Botswana, Namibia, Zambia, Angola, South Africa and Zimbabwe, with an estimated population of 113 000 (Hitchcock et al., 2016).

The San's way of life has always been thought of as based on hunting game and gathering wild edible fruits (Kangira et al., 2019). This traditional, nomadic way of life has dramatically changed in all the seven countries because of displacements and land tenure laws that favour permanent settlements and criminalises or prohibits game hunting. Contact between San communities in the different countries is very minimal; with the exception of few San families in Zimbabwe and Botswana that have maintained contact (Kangira et al., 2019). Years of separation from each other and assimilation into different ethnic communities in the respective countries have meant loss of cultural homogeneity with regards to socio-cultural norms, language, self-identify (name), history and beliefs (Robins, 2001b; Kangira et al., 2019; Phiri et al., 2019). A lot of research has been carried out about the San people in Botswana, Namibia and South Africa, as compared to Zimbabwe where such literature about the Zimbabwean San community is very scanty.

What is certainly known is that in Zimbabwe, the San are mostly found in parts of Tsholotsho district in Matabeleland North, and in parts of Bulilima district in Matabeleland South (Madzudzo, 2001; Hitchcock et al., 2016). As Table 1 above shows, the San in Zimbabwe are estimated to be around 2500, which is about 22% of the total Southern Africa San population. There are more San people in Tsholotsho than in Bulilima district (Hitchcock et al., 2016). The San were moved to their current location in Tsholotsho, away from the Hwange National Park, during the colonial era when it was designated a wild-life area under the Game and Fish Preservation Act of 1929 (Madzudzo, 2001). Their proximity to the Hwange National Game Park, in the extreme southern and western parts of Tsholotsho district, is therefore strategic for hunting-based livelihoods opportunities (Hitchcock, 1999). They live in the outlying parts of the District, towards the national game park, alongside the Ndebele and Kalanga communities (Madzudzo, 2001). With no assets and wealth-derived social power, the San are easily dominated by the Kalanga and the Ndebele ethnic groups and exploited as cheap labour (Kangira et al., 2019).

**Table 1:** Estimated San population in southern Africa by country.

Country	Population estimate
Angola	1 200
Botswana	47 675
Namibia	32 000
South Africa	4 350
Zambia	300
Zimbabwe	2 500

The San community in Zimbabwe identifies itself as the *Tshwa* (meaning a person) (International Labour Organisation, 2013). This name *Tshwa* also depicts the language spoken by the San (Huebschle, 2017). The San people of Zimbabwe are also known as ‘*Abathwa*’ or ‘*Amasili’* in the local Ndebele language and as ‘*Bakhwa’* in the local Kalanga language. The latter groups are themselves referred to by the San people as ‘*Abesintwini*’ (loosely translated of Bantu origin). All the San identity names are perceived by the San as derogatory (Madzudzo, 2001), and indirectly reflect the minority status and the extant asymmetrical power dynamics and socio-economic relations between the San and the dominant Ndebele and Kalanga neighboring ethnic communities. These exert unequal power dynamics and socio-economic relations point to the difficulties that the San community has experienced with regards to integration into the wider society, and in particular, into their neighbouring Ndebele and Kalanga communities (Robins, 2001b). Further, the San's way of living in Zimbabwe, as is the case with other San communities in other countries, has drastically shifted from nomadic, hunting and gathering livelihoods-based activities to permanent agro-pastoralism forms of livelihoods (Kangira et al., 2019). In a fast modernizing and globalizing world, such livelihoods and cultural transformations posit serious challenges for marginalized communities like the San to adapt and sustain livelihoods. It is important therefore to conduct comprehensive studies in order to understand how the twin processes of modernization and globalization is affecting marginalized communities' livelihood asset portfolios and resilience capabilities (Robins, 2001a).

## 2 Literature review (access to education)

Education is considered essential for sustainable development and is a fundamental human right, as stated by article 26 of the Universal Declaration of Human Rights (UN, 2013). There is a widespread consensus on the importance of education for human well-being (Glewwe and Kremmer, 2005). For instance, Sen (1999) argues that education has a direct relevance to the well-being and freedom of people as well as an indirect role through influencing social change and economic production. It is also important to note that Zimbabwe is part of the global community that is obligated to fulfil Sustainable Development Goal 4 which is to ensure inclusive and equitable quality education and promote lifelong learning opportunities for all. The government of Zimbabwe recognizes education as a basic human right and attempts to improve access to education for all its citizens (Ndlovu et al., 2014). The San community are doubtlessly part of the stakeholders that deserve to have unhindered access to quality education like all humanity.

### 2.1 Challenges to education access by San people in Southern Africa

#### 2.1.1 Discrimination

San people's access to education is hindered by discrimination that they suffer from other learners and teachers. For example, in Namibia teasing and bullying of San children in schools where they are a minority is serious and many of them drop out of school while some hide their identities. Teachers also treat San parents and their children in a manner that make them feel alienated in the formal education sector hence they withdraw from school which puts them into intergenerational poverty risk (Cultural Survival International Group, 2015).

#### 2.1.2 Language barriers

Lack of teachers fluent in San language has contributed to poor performance at school by San children. The majority of San children are not taught in their mother language and this makes many children to underperform and some to drop out of school. The Cultural Survival International Group (2015) cites that indigenous children taught in English face language barriers and most of them fail to complete school as they drop out because of challenges they face in trying to understand different concepts taught in the classroom by teachers. The extinction of a language results in the irrecoverable loss of unique cultural knowledge. For the San and any other community, language is the creation and the vector of tradition. They support cultural identity and are essential part of a community heritage (UNESCO, 2017). Furthermore, Africa is the only continent where the children start school using a foreign language. This creates a system where young people look at their native language as a burden rather than as a tool of expression and production (Dahir, 2017).

#### 2.1.3 Hostile school environments

School environment compared to home environment seems to be hostile for many San learners. Tensions between San families and school staff arise if children run away from being disciplined by teachers in Namibia (Newbould (2010), cited in UNICEF (2011). Holsinger and Jacob (2009) narrates that high levels of dropouts of San children in South Africa's San communities are a result of abuse that they suffer from school authorities and other children. Bullying in boarding schools and poor living conditions affects children seriously. Hence San children because of the hostile school environment, fail to access education which contributes to chronic poverty (Holsinger and Jacob, 2009).

#### 2.1.4 Long distances travelled by children to school

According to Anaya (2013), distances that children travel to nearest schools poses serious barrier to education access. Public transport is also unaffordable and transport is irregularly provided, as a result children travel long distances that puts them at risk of being abducted. Furthermore, according to Hays (2011), in Botswana access to education has been hindered by the long distances they travel to nearest schools. Because of long distances, San children drop out of school while some never enrol which poses serious problems to their future and also expose them to child labour.

#### 2.1.5 Poverty

Holsinger and Jacob (2009) argues that San people have received the least attention and worst treatment among all South Africa's ethnic groups. The government have paid less attention to their economic, cultural and political lives. Because of severe financial problems faced by the San people, many San children fail to enrol at school while some drop out and underperform at school. This further indicates that marginalisation affects children's access to education as failure by governments to support minorities result in them failing to send their children to school which robs them of a better future. According to Anaya (2013), poverty makes it absolutely difficult for San children to enrol and complete schooling in Namibia. Although the country ‘s constitution requires that primary education be accessed by all on free of charge, the 2001 Education Act allows schools, to charge fees for school development fund that is used for maintaining school facilities and to improve sport, cultural and education activities. San students are usually turned away from school for failing to pay development fund regardless of the fact that they are supposed to be exempted from paying fees. Since they cannot afford to pay, this results in them dropping out and becoming child labourers.

The San people in Southern Africa do not view education as a driver for economic prosperity. San children across southern Africa drop out levels in government schools are very high (Hays, 2009). According to the Cultural Survival International Group (2015), a difference in education access exists between minorities and other dominant groups. San people's literacy rates are the lowest at 23% while at national level the average is 66% (Cultural Survival International Group, 2015). Furthermore, enrolments both at junior and senior secondary are very low. Most San parents fail to meet school needs like stationery and uniforms and this result in them being bullied by other school children and teachers. San children leave school as a result of hostile environments which they are not used to at home (Hays, 2009).

Hays (2014) narrates that schools are located far away from children's homes and public transport is so costly and not provided regularly in Namibia, hence distances are also one of the drivers for San children ‘s lack of access to education. According to UNICEF (2011), San learners in Namibia are not taught in their home language in primary school and that's another barrier to education. Nudelman (2011) highlights that the government of Botswana although it has thrived to provide free primary education since independence, San people are still left out. San people's lack of access to education include poverty and inequality, and the government's failure to respect their culture, stereotypes from other children and teachers and corporal punishment. Saugestad (2001) narrates that in South Africa, cultural practices like hunting trips, abuse of the San children in the hands of school staff, language barriers all work together to force San children to drop out resulting in poor education outcomes.

Murwira et al. (2016) outlines that San children's education access in Zimbabwe is very low, both among children and adults. Long distances travelled to school and financial problems are the leading factors to high drop outs of the San children. Zhou (2014) ‘s study also indicates that financial barriers affects San people ‘s access to education, as fees are too high for the poor San people and they are not in a position to buy stationery. He further points out that because of secondary school's high fees, parents fail to pay it hence those who complete primary school cannot manage to go to secondary school.

Poverty, geographical location caste, race, ethnicity, livelihood and disability falls under factors that contributes to indigenous people's lack of access to education. Minority people in Turkey since independence have been denied the right to access education (Kaya, 2009). The Minority rights group international (2009), asserts that in Guatemala, indigenous populations live in rural areas and 65 % of men cannot write or read and 65% of women are also illiterate. In Latin America, poverty, child labour and long distances to school are responsible for indigenous people's lack of access to education. Warrilow (2008) highlights that the pastoralist, Maasai and Barbaig of Tanzania's access to education is hindered by lack of facilities and this has resulted in high levels of illiteracy among pastoralists. He further narrates that neglect by the government have prevented the Batwa people of DRC, Rwanda, Uganda and Burundi. Hence this clearly indicates that marginalised communities globally, face serious challenges in accessing education.

## 3 Materials and methods

The study utilised a two-pronged methodological approach that blended a mixed method design which integrated both qualitative and quantitative of data collection. The mixed method design used three main data collection instruments; namely the questionnaire, in-depth interviews and focus group discussions (Teddlie and Yu, 2007; Ndlovu, 2012; Ndlovu and Moyo, 2019). A total of 200 questionnaires were distributed to households in the San communities, with a 25 percent distribution target being deliberately eschewed towards the San households. The San community was targeted through their traditional leaders known as village heads who are of San origin and with the personnel from local NGOs that specifically deal with the community on a daily basis. The five wards in Tsholotsho were selected purposively through the District Education Office and the San traditional leaders who have detailed knowledge on where the San are based geographically and they have interacted with the community for several years. The key informants, which were purposively sampled included the San traditional leaders, District Education Officials, Ward Councillors, Child Protection Agency officials and the Registrar's department in Tsholotsho District. A total of twenty (20) Focus Group Discussions were also conducted in five wards, namely 1, 7, 8, 10 and 11 where the San are predominantly found. Stratified sampling was used to execute focus group discussions in the selected wards. The strata was such that in all wards there were separate groups representing primary school learners (aged between 6 to 12 years), secondary school learners (aged between 14 to 18 years), young parents (aged 23 years–40 years) and mature parents over the age of 40 years. Ethically, the study was approved by the education district office in Tsholotsho and the respondents signed a consent form to participate in the research after the objectives of the study were explained to them. The main objective of the study was to examine the challenges of the San community in accessing primary and secondary education in Tsholotsho district. The data was analysed thematically. The themes that emerged to address the study objective were: poverty, long distance to school, perceived negative attitude towards school and poor infrastructure and compromised quality of learning.

## 4 Results and discussions

This section presents the findings of the study. It examines children's attendance of primary and secondary school as well as their performance. The presentation makes a deliberate effort to compare San community children's performance with those of the neighbouring Kalanga and Ndebele communities. It was noted that the language of instruction in schools in Tsholotsho is Ndebele and Kalanga. The San children are not fluent and skilled in those languages which invariably affects their overall performance in subjects taught. They are excluded. School reports show that Ndebele and Kalanga children outperform learners from the San community. The study generally finds out that attendance of school both at primary and secondary levels is very poor especially amongst the San communities. The Deputy headmaster at Butabubili Primary School aptly summed up the school attendance situation by stating that;

*We are not satisfied with the way children attend school especially those from the San community. The San community has a lot of children at home.* (Butabubili, Primary, School Head KII)

It was also found that teenage pregnancies are a major point of concern in all the communities including the San, Kalanga and Ndebele communities. The discussion that follows below explains in detail the issues raised here.

### 4.1 Causes of non-attendance of school

#### 4.1.1 Long distance

Evidence from both the focus group discussions and key informants in selected villages namely Mtshina 1, Gariya, Nemane, Sithembile and Sakhile showed that long distances to school were preventing the San community from attending primary and secondary school. The respondents were unanimous in their observation that long distances to school discouraged their children from accessing education. The community noted the fact that their children took a long time to reach school. In relation to that, three respondents in a focus group discussion said,

*My children walk long distances to school and hence I do not get time with them to know what is happening at school as they come home very late and they do not get time to read as well.* (Sakhile Female FGD)

*The authorities have never prioritised us as the San community from time immemorial. Schools are built far away from our homes and close to the Ndebele and Kalanga communities. The government does not care about us. Simply as that. (Sithembile Male FGD)*

*The San community is a nomadic society. They don't stay in one place for a long time. They are always moving even to neighbouring countries like Botswana. Even if a school is constructed close to them, they dessert the school and move further from it. They do not want to be mingled with other tribes. Their culture and traditions are different from the rest of the communities. You will never understand these people. (Gariya, Key informant interview)*

In a different village called Mtshina, similar sentiments were echoed that long distances children travel to school demoralizes most of them. It emanated from this focus group discussion that children doing early child education return home before reaching the school due to the long distances they have to travel. In Mtshina it was observed that the time taken to reach the nearest school was approximately 2 hours. The following respondents noted that:

*The distance is too long to get to school especially for the younger children. If they leave home at 6am they get to school around 8am. So they are also usually late at school.* (Mtshina Female FGD)

*Both primary and secondary schools are far from us. Khumbula secondary school is eight hours walk from where we stay. The fees they charge is beyond us. I understand the school fees is $500. It's been years ever since I held $10. What more of these figure the school is demanding. Our children would rather stay at home and do domestic chores than waste their time going to these far schools which we cannot even afford.* (Sakhile Male FGD)

The Deputy Headmaster at Butabubili Primary School raised similar concerns about long distances being travelled by students to school. She noted that the school was drawing pupils from very distant places. She pointed out that:

*The challenge is that the schools are very far, children walk long distances to school that even adults cannot handle. Sikhente is 20km away and Mbiriya is 30km away and the children from there come as far as this school.* (Butabubili Primary School KII)

Apart from the concerns raised by their parents over long distances to travel to school, the children themselves verified and validated these claims in focus group discussions. In a focus group discussion for pupils at Phelandaba Primary School, pupils noted that long distances to school were a major reason for school dropouts. For example it was noted that:

*Boy XYZ left school last week; he has been seen herding the headman's cattle. We can't ask him to come to school because he stays very far. We were told that he was complaining that school is far and his father said he should find a job.* (Phelandaba Primary School Boys FGD)

Pupils at Phelandaba Primary School pointed out that children who travel long distances to school were affected by exhaustion and hunger and they found it difficult to concentrate during lessons. It emerged as well from the respondents that those who attend school from far places often sleep in class due to fatigue and in some cases they faint or collapse.

**Figure 1:**
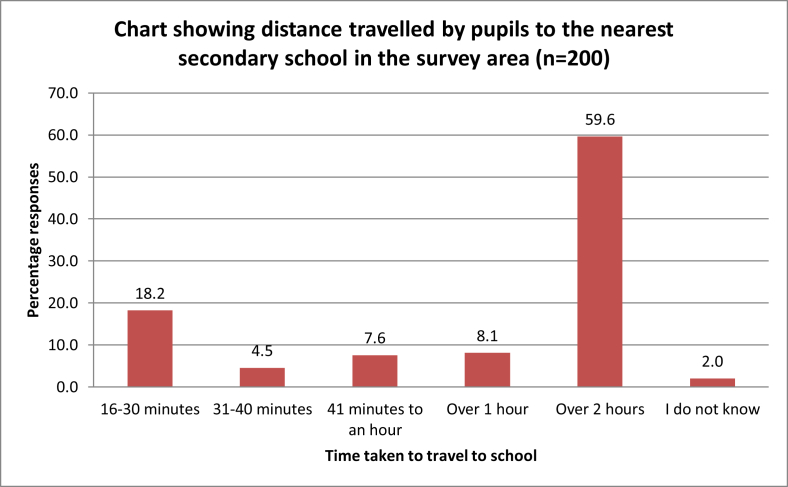
Distance travelled by pupils to nearest secondary school (Source: Survey data, 2016).

The results from Figure 1 show that 59.6% of the pupils travel over 2 h to get to the nearest secondary school, while 8.1% travel over an hour, 7.6 % travel between 41 min to an hour, 4.5 % of the students travel 31–40 min, 18.2 % travel 16–30 min while 2% do not know how much time they travel. However, it is clear from these results that time taken to travel to secondary school affects the majority of the pupils.

Table 2, below reveals that, from the 11 sampled villages, four villages (Gariya, Sakhile, Sithembile and Sibambene) had the highest number of pupils that travel over 2 h to the nearest secondary school. It was observed that Nemane was the only village with the nearest secondary school where pupils travel for less than 30 min. Mtshina, Mpilo and Thabisa village has a significant number of pupils travelling for more than an hour. In general, about 60% of the surveyed households had children walking over 2 h to the nearest secondary school. Table 2 below shows the general distances travelled by village.

**Table 2:** Distance travelled to nearest secondary school by village.

		In this area how long does it take to get to the nearest secondary school? (n = 200)					Total
		16–30 min	31–40 min	41 min to an hour	Over 1 h	Over 2 h	
Village	Gariya	7.8%	2.0%	2.0%		88.2%	100.0%
	Muzimunye	11.8%			17.6%	70.6%	100.0%
	Thula	76.9%	15.4%	7.7%			100.0%
	Mtshina	6.7%	6.7%	43.3%	23.3%	20.0%	100.0%
	Mpilo				22.2%	77.8%	100.0%
	Sakhile					100.0%	100.0%
	Sifulasengwe		50.0%			50.0%	100.0%
	Nemane	100.0%					100.0%
	Sithembile	9.1%	4.5%		4.5%	81.8%	100.0%
	Thabisa	22.2%			11.1%	66.7%	100.0%
	Sibambene				20.0%	80.0%	100.0%
Total		17.5%	4.4%	8.2%	8.7%	61.2%	100.0%

Focus group discussants amongst the youths pointed out that they would happily attend school if there were available schools closer home. For instance, in Sakhile village, it was revealed that the nearest secondary school is Khumbula, which is 42km away from them. Access to secondary school is therefore impossible given the geographical distance being mentioned. Pupils who completed primary school at Sakhile either had to go to boarding school or find a school in Tsholotsho urban or Bulawayo. Those children whose parents could not have enough money to find alternative schools eventually dropped out of school without getting to secondary school level. Therefore, the parents emphasized the need for schools to be constructed closer to their homesteads.

**Figure 2:**
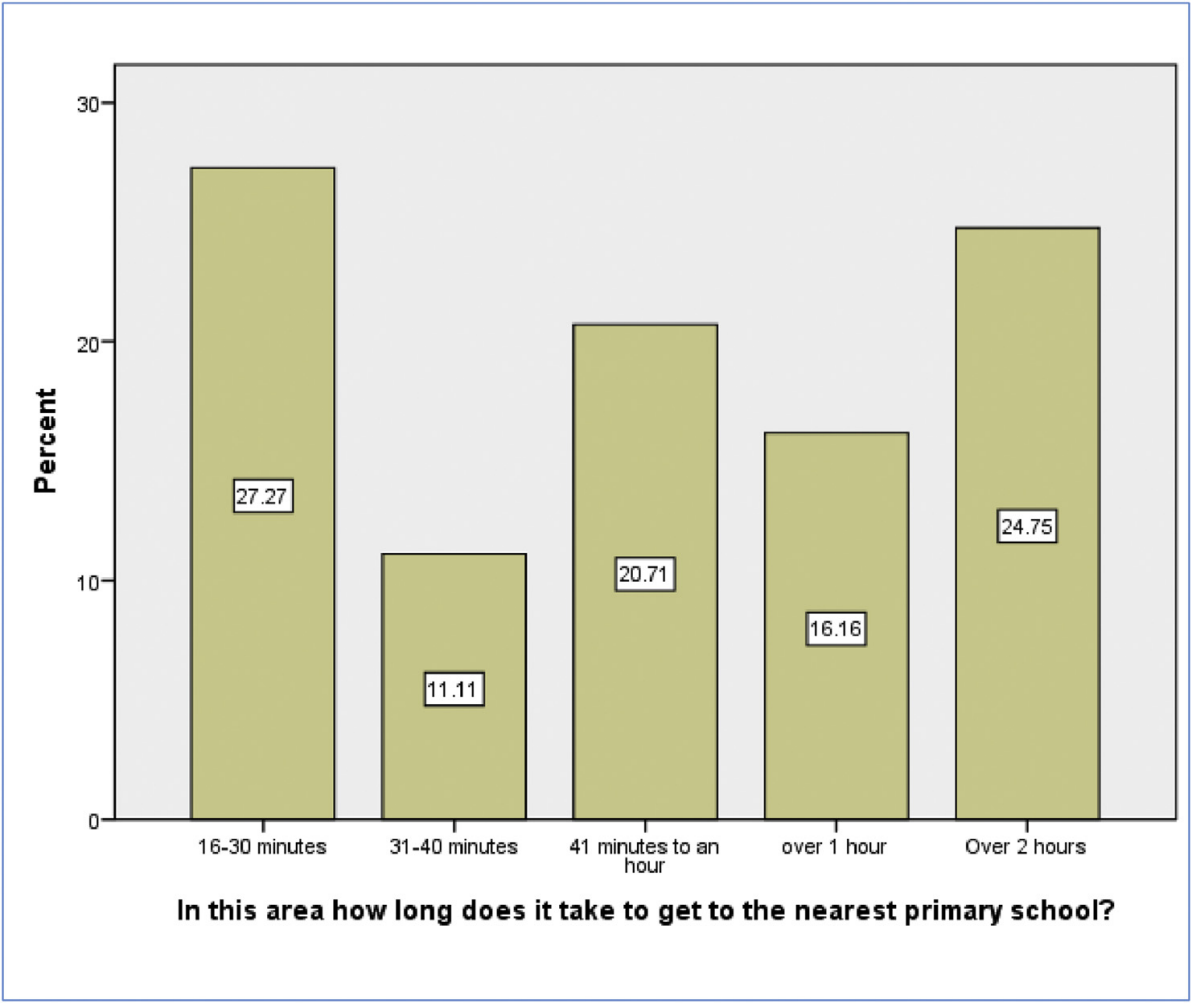
Time taken to get to the nearest primary school (Source: Survey data, 2016).

Figure 2 shows that 24.74% of the pupils travel over 2 h to get to the nearest primary school. 16.16% of the pupils take over an hour, 20.71 % take 41 min to an hour, 11.11% take 31–40 min and 27.27% take 16–30 min. In this particular case, distance travelled to primary schools is lesser compared to secondary schools, although it still remains relatively higher in some places.

Table 3 indicates that villages like Mtshina, Mpilo, Sakhile and Sibambene have the greater percentage of pupils who travel over 2 h respectively. On the other hand, Nemane and Thula village have the greatest percentage of pupils who travel between 16-30 min.

**Table 3:** Table showing time taken to get to primary school by village.

		In this area how long does it take to get to the nearest primary school?					Total
		16–30 min	31–40 min	41 min to an hour	over 1 h	Over 2 h	
Village	Gariya	25.5%	13.7%	41.2%	9.8%	9.8%	100.0%
	Muzimunye	14.3%	9.5%	14.3%	33.3%	28.6%	100.0%
	Thula	84.6%	15.4%				100.0%
	Mtshina	13.3%		23.3%	13.3%	50.0%	100.0%
	Mpilo	22.2%	11.1%		22.2%	44.4%	100.0%
	Sakhile	20.0%	20.0%	20.0%		40.0%	100.0%
	Sifulasengwe	42.9%	28.6%		28.6%		100.0%
	Nemane	87.5%	12.5%				100.0%
	Sithembile	19.2%	11.5%	11.5%	19.2%	38.5%	100.0%
	Thabisa	22.2%	5.6%	16.7%	38.9%	16.7%	100.0%
	Sibambene		20.0%	40.0%		40.0%	100.0%
Total		27.3%	11.1%	20.7%	16.2%	24.7%	100.0%

The narratives from the focus group discussions and key informant interviews show a deeply entrenched systematic marginalisation of the San community with regards to access to primary and secondary education. Long distances militant against them in accessing education. No African country (Zimbabwe included) has achieved universal primary education (UNESCO, 2017; AAI, 2015). About half of all out of school African children will never step foot in a school in their lifetime. The government of Zimbabwe has never made concerted effort to address the construction of schools near the San community. The Tonga people in Binga district of Zimbabwe also experience the same predicament (Dziva and Dube, 2014; Makoni et al., 2012). Since 1980 when Zimbabwe got its independence, there is no single school that has been solely constructed to address the needs of the San community in view of the long distances they are facing. This paper argues that this institutionalised marginalisation and calculated social exclusion will continue as things stand. The Sustainable Development Goal 4 of ensuring inclusive and equitable quality education and promoting lifelong learning opportunities for all will remain a pipe dream in Tsholotsho.

#### 4.1.2 Poverty

The high poverty levels amongst the San communities make it difficult for them to afford to send their children to school. This issue was raised in ethnographic observations, focus group discussions and key informants. Poverty directly affected parents’ ability to pay school fees, purchase school uniforms, exercise books, pens, pencils and food for children. A general feeling of despair and indifference was expressed in focus group discussions with community members as they felt that as long as they could not raise enough money their children could not attend school. Poverty inhibits them from providing adequate resources for their children to attend school.

The focus group participants were quick to point out that their children are sitting at home when asked whether their children are attending school due to fees challenges. It was revealed from one of the focus group discussions that:

*Some of us can only afford to send our children to primary schools, but we do not meet the expense of secondary school fees so the children end up dropping out of school even if the child is intelligent (Phelandaba FGD Females).*

*School fees is a challenge, we don't have the money, as we speak most of the children are at home, all those who have not paid school fees were sent back home this week. As we speak now, they were chased away from school for non-payment of school fees. Its four days now since they were chased from school. We have taken our cocks to pay the fees but now but how will the chickens reproduce without cocks?* (Sanqinyane Male FGD)

*I am a very old poor woman and taking care of four orphans in this house. My strength has been exhausted. Where do you think I get the money to buy books, school uniforms and pay fees? We are struggling to get a decent meal daily. Going to school is for children with rich parents. (Mtshina Female FGD)*

Similar sentiments were expressed in Gariya focus group discussions. One respondent remarked that;

*Failure to purchase stationery and uniforms for our children is one of the reasons why they do not attend school*. (Gariya Male FGD)

While poverty affected most pupils from the San communities, respondents indicated that children who were orphans were in a particularly difficult position with regards to poverty and its effects on school attendance. It was pointed out that orphans were mostly under the care of grandparents who had no capacity to raise the required school fees and other school expenses. Some of the grandparents who were guardians to the orphans were well over 70 years and they were no longer economically active due to age. In fact, they were struggling to feed the grand children they look after. (Sakhile village Female FGD).

Parents were reportedly adopting various methods to cope with poverty as regards the education of their children. In some instances parents would take their children to primary school only. Secondary school was said to be more expensive according to the respondents. For instance in the Nemane Women's Focus Group Discussion, one participant pointed out that;

*Some of us can only afford to send our children to primary schools, but we do not have the money required for secondary school fees so the children end up dropping out of school even if the child is intelligent.* (Nemane Female FGD)

It became apparent from the discussions that most parents were willing to send their children for secondary education but they were limited by finances. It was revealed that some students excel at primary level (grade 7) but could not proceed to the next level due to fees. Youths in Gariya village corroborated with this revelation when they postulated that from their generation they were unable to complete primary education and had to drop out as they purported that from a ratio of 4/12 of us ended up at primary level of which 2/12 only proceeded to secondary level.

Besides the inability to pay school fees, poverty also affected the physical well-being of many pupils. It emerged from the respondents that going to school for poverty stricken school children was a challenge in many ways. In most instances, they walked bare-footed which exposed their feet to the hot ground during summer and frosty conditions during winter. Moreover, they were also not comfortable to go to school because they would not be well presentable due to lack of bathing soap and body lotion. Their peers in school often discriminated and isolated San community children on the basis that they “smell”. Consequently, the lack of resources caused them to shy away from school to avoid ridicule and mockery from other pupils. One of the San respondents noted that:

*When our children go to school and get there, they are ridiculed by the other children. When they sit together on a bench they say to them, can you please move to the far end, you smell.* (Mtshina Female FGD)

With regards to poverty, it has been reported that the San community suffer from the scourge more severely than the neighbouring Ndebele/Khalanga tribes (Hitchcock et al., 2016; Phiri et al., 2019). Consequently, the need for food overrides the need for education. As has been reported in other studies, the San community survives by working in the farms of other wealthier tribes due to poverty and have no luxury to attend school (Mumpande, 2006; Muzondidya and Gatsheni, 2007). The former president of Zimbabwe, Robert Mugabe was once quoted saying: ‘The San community likes the bush and meat than we do’. This study gathered that the pressing need for the community is access to food more than the access to education. Once the food aspect of their lives is dealt with, the drive to attend school can easily be solved with. Free primary education has been a mere rhetoric and has not been fully implemented. This is a serious bottleneck to the attainment of education for the San community. This paper brings to the fore that the needs of the San are overlooked and suppressed, a concept popularly known as seen but not heard (Lansdown, 2001; Maunden, 2002).

#### 4.1.3 Perceived negative attitudes towards education

An interaction with various stakeholders including school authorities showed that the San were generally perceived as not being interested in modernisation and education. San community parent were thought to have negative attitude towards school and not to be see the value of education in their children. It was believed that some parents were denying their children the opportunity to attend school. One of the key informants from the education sector remarked that:

*The problem is with the San parents. They don't value education and they don't provide food and clothes for the children. Even when we provide uniforms and food for them, the parents don't come to work here at school. The children are willing to learn and are very intelligent but the parents are dragging their feet.* (Key Informant name withheld)

*The San community resists civilisation and modernisation. I have worked here for more than twenty years now. We have tried to convince them of the importance of education to no avail. We even constructed a primary school close to them and they relocated to another village. I don't really know what they want. They are just a nuisance. None of their children ever finish their secondary school.* (Key Informant name withheld)

However, the authenticity of this stereotype is difficult to ascertain in the presence of a multiplicity of factors that affect the ability of San households to send their children to school. It seems to us that the stereotype might not be entirely founded on facts but rather on a misunderstanding of the status of the San people and the socio-economic dynamics that they face in an evolving modern world.

According to a newspaper report in 2013, the then Minister of Education, David Coltart noted that “The San are a marginalised community and we will have to assist them with policy implementation to address their problems on education. Unfortunately awareness of the marginalisation does not translate to addressing the challenge. The magnitude of the challenges experienced by the San community are documented (Kangira et al., 2019; Hays, 2011). This paper infers that there is no political will to accommodate and assist the San community in their uniqueness.

#### 4.1.4 Infrastructure and quality of learning

There was concerned in most of the surveyed wards about the state and quality of education. The main challenges ranged from inadequate infrastructure to a lack of commitment by teachers. Youths from Gariya who participated in a focus group discussion indicated that their siblings had no classrooms and they were having their lessons under trees. They also revealed that where there are classrooms, pupils fought over furniture. They therefore were forced to go to school earlier in order to secure a chair and a desk.

Parents also raised concerns from the focus group discussions that there was insufficient teacher's accommodation in the primary schools which affects the quality of education. This problem reportedly caused teachers to be frequently changing schools and that meant children usually did not have the same teachers throughout the year. This inconsistency affected the performance of pupils in a negative way. As one respondent indicated in the Women Focus Group Discussion in Nemane village:

*We do not get enough qualified teachers because living quarters are overcrowded. As such, children are not taught well because they are given adequate and enough education as a teacher who reported for duty the last term would have gone and is replaced by another, whilst a child is supposed to get one teacher a year so that the child gets continuous and an enhanced teaching. (Nemane Female FGD)*

In addition, it was also reported that schools have shortage of teachers which makes learning difficult. For instance, grade four and five pupils were in some instances made to learn from the same class. The parents from Sakhile village indicated that they were not happy with the status quo. Another sentiment that was aired concerned the fact that teachers were perceived not to be serious about their work. It was revealed that;

*There is no seriousness in the way teachers work, some spend a week away from work and no one cares. Ministry of Education no longer supervises these teachers may be it because we are in the rural areas. (Sanqinyane Male FGD)*

## 5 Conclusion

The study established that the San community faces a plethora of challenges in their attempt to access primary and secondary education. The embedded stereotypes and prejudices of neighbouring ethnic groups like the Ndebele and Kalanga complicate and compound the situation. It was also revealed from the study that abject poverty has made it difficult for the San community to get access to formal education. Survival takes priority over every other activity.

The paper also revealed problems associated with long distances travelled to get to school over and above the poor infrastructural facilities and low morale of teachers. The study recommends that provision of bicycles could assist to make travel easier for school children who travel in excess of 2 h to reach their schools. Enhancement of livelihoods diversification could help to support parent raise school fees for their children. Parents should be taught the importance and value of education. Programming should focus on showing the importance of education to both children and parents in the whole survey area as there is a general attitude of disinterest in education.

## Declarations

### Author contribution statement

K. Phiri: Conceived and designed the experiments; Performed the experiments; Analyzed and interpreted the data; Wrote the paper.

S. Ndlovu: Conceived and designed the experiments; Performed the experiments.

D. Nyathi: Performed the experiments.

C. Ncube: Performed the experiments; Contributed reagents, materials, analysis tools or data.

T. Dube: Performed the experiments; Analyzed and interpreted the data; Contributed reagents, materials, analysis tools or data.

N. Tshuma: Performed the experiments; Contributed reagents, materials, analysis tools or data.

### Funding statement

This research did not receive any specific grant from funding agencies in the public, commercial, or not-for-profit sectors.

### Competing interest statement

The authors declare no conflict of interest.

### Additional information

No additional information is available for this paper.
